# Safety and efficacy of Amplatzer duct occluder II and konar-MF™ VSD occluder in the closure of perimembranous ventricular septal defects in children weighing less than 10 kg

**DOI:** 10.3389/fcvm.2023.1255808

**Published:** 2023-11-29

**Authors:** Kaan Yildiz, Nazmi Narin, Sedef Oksuz, Rahmi Ozdemir, Ozge Pamukcu, Ali Baykan, Abdullah Ozyurt, Sedat Bagli, Rasit Aktas, Ikbal Nur Safak, Muhammed Akif Atlan, Yunus Sezer Bayam, Cem Karadeniz

**Affiliations:** ^1^ Department of Pediatric Cardiology, SBU Tepecik Training and Research Hospital, Izmir, Türkiye; ^2^ Department of Pediatric Cardiology Izmir, Katip Celebi University Faculty of Medicine, Izmir, Türkiye; ^3^ Department of Pediatric Cardiology, Erciyes University Faculty of Medicine, Kayseri, Türkiye; ^4^ Department of Pediatric Cardiology, Istinye University Faculty of Medicine, Istanbul, Türkiye; ^5^ Department of Pediatrics, SBU Tepecik Training and Research Hospital, Izmir, Türkiye

**Keywords:** ventricular septal defect, transcatheter closure, weight >10 kg, Amplatzer duct, KONAR-multi functional occluder

## Abstract

**Introduction:**

Device closure of perimembranous ventricular septal defects (pmVSD) is a successful off-label treatment alternative. We aim to report and compare the outcomes of pmVSD closure in children weighing less than 10 kg using Amplatzer Duct Occluder II (ADOII) and Konar-MF VSD Occluder (MFO) devices.

**Methods:**

Retrospective clinical data review of 52 children with hemodynamically significant pmVSD, and sent for transcatheter closure using ADOII and MFO, between January 2018 and January 2023. Baseline, procedural, and follow-up data were compared according to the implanted device

**Results:**

ADOII devices were implanted in 22 children with a median age of 11 months (IQR, 4.1–14.7) and weight of 7.4 kg (IQR, 2.7–9.7). MFO devices were implanted in 30 children with a median age of 11 months (IQR, 4.8–16.6) and weight of 8 kg (IQR, 4.1–9.6). ADOII were implanted (retrograde, 68.1%) in defects with a median left ventricular diameter of 4.6 mm (IQR, 3.8–5.7) and right ventricular diameter of 3.5 mm (IQR, 3.1–4.9) while MFO were implanted (antegrade, 63.3%) in defects with a median left ventricular diameter of 7 mm (IQR, 5.2–11.3) (*p* > 0.05) and right ventricular diameter of 5 mm (IQR, 2.0, 3.5–6.2) (*p* < 0.05). The procedural and fluoroscopy times were shorter with the MFO device (*p* < 0.05). On a median follow-up of 41.2 months (IQR, 19.7–49.3), valvular insufficiency was not observed. One 13-month-old child (6.3 kg) with ADOII developed a complete atrioventricular heart block (CAVB) six months postoperative and required pacemaker implantation. One 11-month-old child (5.9 kg) with MFO developed a CAVB 3 days postoperative and the device was removed. At 6 months post-procedure, only one child with MFO still experiences a minor residual shunt. There was one arterio-venous fistula that resolved spontaneously.

**Conclusion:**

Both the MFO and ADOII are effective closure devices in appropriately selected pmVSDs. CAVB can occur with both devices. The MFO is inherently advantageous for defects larger than 6 mm and subaortic rims smaller than 3 mm. In the literature, our series represents the first study comparing the mid-term outcomes of MFO and ADOII devices in children weighing less than 10 kg.

## Introduction

Perimembranous ventricular septal defects (pmVSD) are the most common type of VSD, accounting for approximately 80% of all ventricular septal defects (1). In recent years with the development of the new devices transcatheter closure of VSD becomes an alternative treatment option to open heart surgery (2–5). Although primarily used for closing patent ductus arteriosus (PDA) with nitinol wire mesh, the Amplatzer Duct Occluder II (ADOII) device (Abbott Cardiovascular, MN, USA) is also safely employed for treating pmVSD. There are studies available advocating that the flexible disk of the device can significantly reduce the risk of complete atrioventricular block (CAVB) (6–8). On the other hand, the KONAR-MF™ VSD occluder (MFO) device (Lifetech, Shenzhen, China) is preferred primarily for children with large pmVSD due to its soft and flexible structure, from both sides used (9, 10). In children weighing less than 10 kg with pmVSD, appropriate children selection and proper choice of device for transcatheter closure help minimize complications, such as device embolization, residual shunt, valve insufficiency, and arrhythmias (11–13).

This study aims to retrospectively compare the mid-term safety and efficacy of the MFO device and the ADOII device in children weighing less than 10 kg who underwent transcatheter closure for pmVSD.

## Patients and method

**Figure 1:**
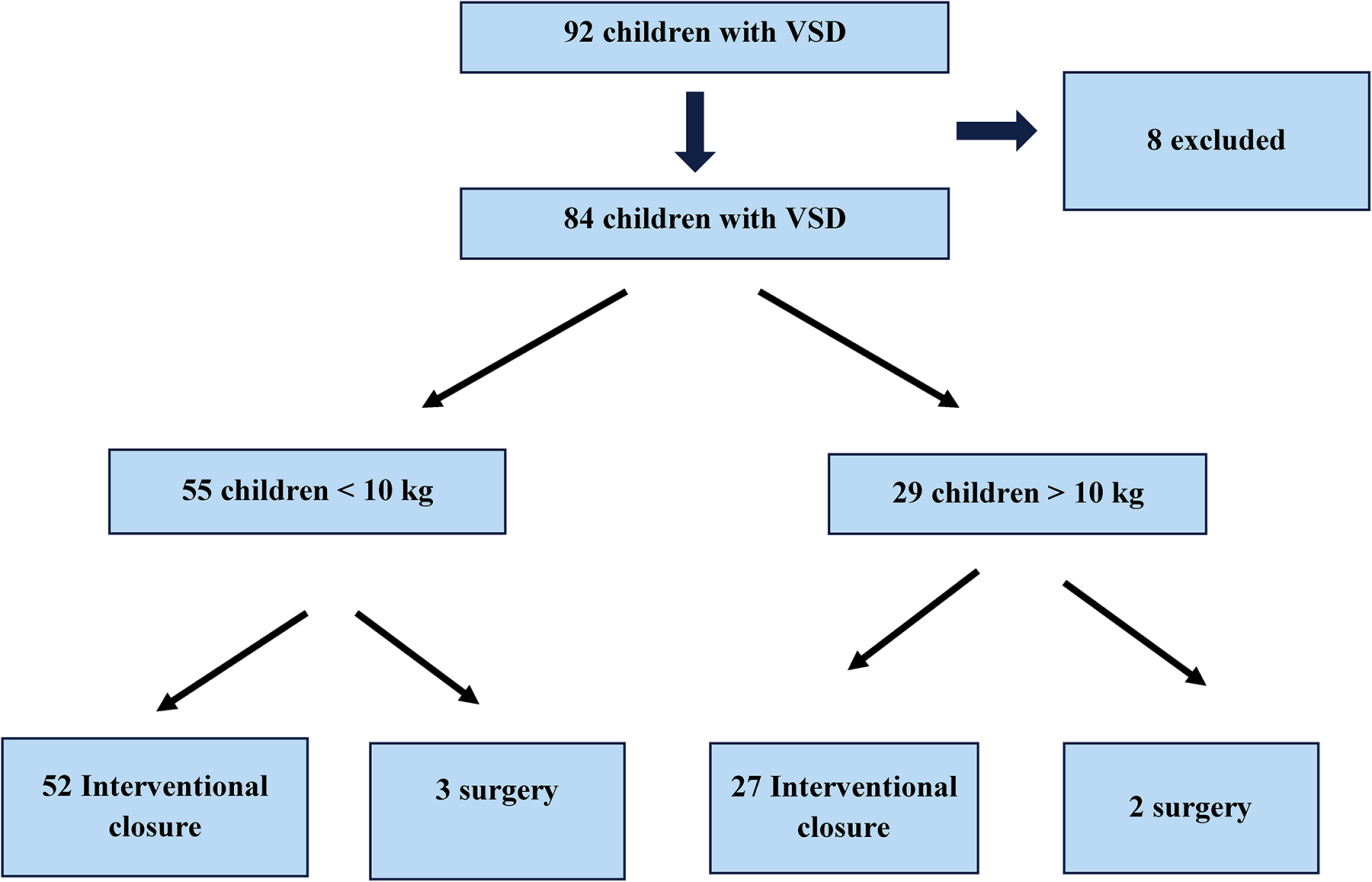
Study chart.

We performed a retrospective clinical data review of 52 children with hemodynamically significant pmVSD and sent for transcatheter closure using ADOII (*n *= 22) and MFO (*n* = 30), at our institutions between January 2018 and January 2023. We divided the children into 2 groups according to the implanted device and compared the baseline, procedural, and follow-up data (Figure 1).

All procedures contributing to this work comply with the ethical standards of the relevant national guidelines on human experimentation, and with the Helsinki Declaration of 1975, as revised in 2008. Approval from the institutional review board was obtained. Written informed consent was signed by the patients or their legal guardians to perform the procedure and to use their clinical records for eventual publication.

### Inclusion criteria

Children who underwent percutaneous pmVSD closure had a clinically significant left-to-right shunt with left heart volume overload. We defined volume overload as echocardiographic LV end-diastolic diameter (LVEDD) Z-score ≥2.0 (14). Indications for closure included: heart failure unresponsive to medications, a cardiothoracic ratio >0.55 on chest x-ray, recurrent respiratory tract infections, and growth failure unrelated to malnutrition. All patients had detailed TTE before the closure procedure to assess the pmVSD location, morphology, size, and hemodynamic relevance. We recorded the proximity of the defect to the aortic and tricuspid valves and the degree of aortic or tricuspid valve insufficiency. The sub-aortic rim (SAR) was measured as the distance from the aortic valve (AoV) annulus to the upper margin of the color flow across the pmVSD using four views (parasternal long-axis view, apical 3-chambers, apical 5-chambers, and subcostal LV-to-Aorta). Children without SAR deficiency (≤2.5 mm for MFO and ≤3 mm for ADOII), mild aortic valve prolapse, and aortic regurgitation (AR) not exceeding mild grade were deemed suitable for transcatheter closure.

### Exclusion criteria

We excluded children with SAR deficiency (≤2.5 mm for MFO and ≤3 mm for ADOII), aortic insufficiency exceeding a mild degree, left or right ventricular outflow tract obstruction, mean PA pressure exceeding 20 mmHg, additional cardiac anomalies requiring surgery, and children whose parents did not provide consent for transcatheter closure.

### Device selection protocol

ADO-II devices were implanted in defects with an LV entry diameter <6 mm, an RV exit diameter ≤5.5 mm, and a SAR ≥ 3 mm. In defects without an aneurysm, ADO-II was selected 2–3 mm larger than the largest defect size. On the other hand, in defects with large aneurysm sacs, the ADO-II was selected equal to or slightly larger than the size of the aneurysm. MFO devices were implanted in defects with LV entry diameter >6 mm and SAR > 2.5 mm. MFO device was selected 1–2 mm larger than the RV diameter or equal to or 1 mm larger than the LV diameter. In the presence of aneurysmal tissue on the right ventricular side, the device was placed within the aneurysm sac to minimize contact with the aortic valve.

### Procedure

Closure procedures were performed under general anesthesia TTE and fluoroscopy, using the antegrade or retrograde approaches as previously described in detail (15, 16). Baseline LV angiograms were obtained in RAO 60°/LAO 30° or RAO 45°/LAO 45° angles, depending on the defect location. We selected the target diameter as the largest defect diameter measured on TTE and angiography. Device selection was made according to the anatomy and mainly operator preference. The defect was crossed from the LV side and closed subsequently according to the chosen approach. In all cases, we evaluated the device placement, the presence of significant residual shunting, and aortic and tricuspid valve insufficiency through LV injection and TTE, before device release.

#### Follow-up protocol

Our team monitored the children using continuous ECG monitoring for the first 24 h after the procedure to detect any post-procedural arrhythmias. We conducted follow-ups comprising clinical examination, ECG, and TTE before discharge (the day after the procedure), at the 4th week, 3rd month, 6th month, and 1st year after device closure. We prescribed aspirin (3–5 mg/kg/day orally) for 6 months to uncomplicated children and discontinued antibiotic prophylaxis for bacterial endocarditis at the 6th month in children without residual shunting.

### Statistical analysis

Statistical analyses were performed using SPSS, Version 26.0 (IBM, Armonk, NY, USA). Categorical variables were reported as frequency and percentage and continuous variables were represented as median with interquartile range (IQR). The normality of measurements was assessed using the Shapiro–Wilk test. Statistical analyses for continuous variables were conducted using Mann–Whitney *U* and by *χ*^2^ test and Fisher's exact test for categorical variables as appropriate. A *p*-value <0.05 was considered statistically significant. All reported *p*-values are two-sided.

## Results

### Patients

ADOII devices were implanted in 22 children with a median age of 11 months (IQR, 4.1–14.7) and weight of 7.4 kg (IQR, 2.7–9.7). MFO devices were implanted in 30 children with a median age of 11 months (IQR, 4.8–16.6) and weight of 8 kg (IQR, 4.1–9.6). The demographic data and interventional parameters of the children are presented in Table 1.

**Table 1:** Demographic, echocardiographic characteristics and procedural data.

	Total, *n* = 52	ADO II, *n* = 22 (42.3%)	MFO, *n* = 30 (57.7%)	*p*-value
Gender				
Male	17 (32.7%)	6 (27.2%)	11 (36.6%)	0.32
Female	35 (67.3%)	16 (72.8%)	19 (63.4%)	0.41
Age (months), median (IQR)	10.8 (4.3–15.2)	11 (4.1–14.7)	11 (4.8–16.6)	0.81
Weight (kg), median (IQR)	7.6 (3.7–9.8)	7.4 (2.7–9.7)	8 (4.1–9.6)	0.83
Associated CHD, *n* (%)	9 (17.3)	4 (18.1)	5 (16.6)	0.13
Concomitant interventional procedure	2	*n* = 1 closure ASD	*n* = 1 PBV	–
Down syndrome	2	1	1	–
TTE findings				
RV size of VSD (mm), median (IQR)	4.2 (3.7–5.4)	3.5 (3.1–4.9)	5.0 (3.5–6.2)	**0.008**
LV size of VSD (mm), median (IQR)	5.6 (4.9–11.3)	4.6 (3.8–5.7)	7.0 (5.2–11.3)	0.065
Aneurysm (*n*, %)	17 (32.6)	8 (36.6)	9 (30)	0.57
SAR (mm), median (IQR)	3.1 (2.1–4.9)	3.8 (2.8–4.7)	2.9 (1.9–5.1)	0.38
Qp/Qs, median (IQR)	2.3 (0.83–2.8)	2.0 (0.81–2.5)	2.4 (0.92–2.8)	**0.029**
Mean pulmonary artery pressure (mmHg) median (IQR)	23 (9.2–26.3)	20 (0.5–25.7)	25 (8.2–29.4)	0.49
Techniques utilized, *n* (%)				
Antegrade	26 (50%)	7 (31.8%)	19 (63.3%)	0.47
Retrograde	26 (50%)	15 (68.2%)	11 (36.7%)	0.36
Device diameter (mm), median (IQR)	5.2 (3.1–11.9)	4.9 (3.2–5.7)	5.4 (3.5–11.8)	0.63
Fluoroscopy time (min), median (IQR)	27.4 (18.2–28.1)	29.5 (19.3–34.5)	19.2 (10.2–28.3)	**0.039**
Total DAP (Gy.cm^2^), median (IQR)	8.8 (7.9–13.5)	10.6 (9.6–12.7)	7.8 (9.8–13.2)	0.195
Procedural success, *n* (%)	50 (96.1)	21 (95.4)	29 (96.6)	0.57
Procedural time (min), median (IQR)	59.2 (32.1–54.3)	61.5 (37.5–59.8)	55.7 (30.6–53.8)	0.53
Device embolization, *n* (%)	1 (1.9)	1 (4.5)	0.0	--
Residual shunt in the first 24 h, *n* (%)	8 (15.3)	2 (9)	6 (20)	**0.042**
Residual shunt at 6 months, *n* (%)	1 (1.9)	0.0	1 (3.4)	0.78
Follow-up duration (months), median (IQR)	41.2 (19.7–49.3)	42.8 (18.9–44.2)	38.7 (18.6–47.5)	0.59

ADOII, Amplatzer™ duct occluder II; MFO, KONAR-MF™ VSD occluder; DAP, dose area product; IQR, interquartile range; LV, left ventricle; RV, right ventricle; TTE, transthoracic echocardiography; SAR, subaortic rim; CHD, congenital heart disease; ASD, atrial septal defect; PBV, pulmonary balloon valvuloplasty.

### Procedure

ADOII were implanted (retrograde, 68.1%) in defects with a median left ventricular diameter of 4.6 mm (IQR, 3.8–5.7) and right ventricular diameter of 3.5 mm (IQR, 3.1–4.9) while MFO were implanted (antegrade, 63.3%) in defects with a median left ventricular diameter of 7 mm (IQR, 5.2–11.3) (*p *> 0.05) and right ventricular diameter of 5 mm (IQR, 3.5–6.2) (*p* < 0.05). The procedural and fluoroscopy times were shorter with the MFO device (*p* < 0.05). The procedure and fluoroscopy times were shorter with the MFO device (*p* < 0.05).

### Follow-up

The median follow-up period in our study was 41.2 months (IQR, 19.7–49.3). Early mild residual shunts were less frequent in the ADOII group compared to the MFO group at 6- and 12-month follow-ups (*p* < 0.05). At 6 months post-procedure, only one child in the MFO group exhibited a hemodynamically insignificant residual shunt. Valvular insufficiency was not observed during long-term follow-up after the procedure. One 13-month-old child (6.3 kg) with ADOII developed a complete CAVB six months postoperative and required pacemaker implantation. One 11-month-old child (5.9 kg) with MFO developed a CAVB 3 days postoperative and the device was removed (Table 2).

**Table 2:** Major and minor complications.

Major complications	Total 1/52 (5.7%)	ADO II, *n *= 22 (42.3%)	MFO, *n* = 30 (57.7%)
Valve injury	0	0	0
CAVB	2	1	1
LBBB	1 (Transient LBBB)	0	1 (Transient LBBB)
Ventricular perforation	0	0	0
Device embolization	1	1	0
Thromboembolism	0	0	0
Minor complications	Total 1/52 (1.9%)		
Transient loss of pulse	0	0	0
New onset aortic regurgitation	0	0	0
New onset RBBB	0	0	0
Mild tricuspid regurgitation	0	0	0
Arteriovenous fistula	1	0	1

ADOII, amplatzer^™^ duct occluder II; MFO, KONAR-MF™ VSD occluder; CAVB, complete atrioventricular block; LBBB, left bundle branch block; RBBB, right bundle branch block.

All cases with inadequate weight gain exhibited rapid weight gain during follow-up. The increased LV end-diastolic diameter regressed to age-appropriate normal Z scores starting from the third month.

Isolated VSD was present in 81.2% of the children. In one child, closure of both atrial septal defect and PDA was performed in the same session, while another child with pulmonary stenosis underwent pulmonary balloon valvuloplasty. Two cases were diagnosed with Down syndrome.

## Discussion

Transcatheter closure of hemodynamically significant pmVSDs in young children has gained more prominence in the past decade, owing to the development of new devices and advancements in operator skills (17–19). The closure of VSDs through device placement has become a frequently conducted procedure, yielding excellent outcomes. High success rates and low complication rates are crucial in terms of selecting the appropriate device and managing potential intra and post-procedural situations. Both ADOII and MFO devices have provided significant advantages over other devices in transcatheter closure, thanks to their flexibility, ease of application, and small delivery systems. Due to variable anatomical morphology and the complexity of the manipulation process, transcatheter VSD closure in children weighing less than 10 kg is technically challenging and requires the expertise of an experienced operator to mitigate the high risk of complications (4, 11–13).

### Technical challenges

ADOII, designed for the closure of small-sized PDAs, offers a more flexible profile compared to conventional double-disc devices made of nitinol mesh wire (8, 20). In our study, especially for defects smaller than 6 mm, ADOII was preferred when SAR was >3 mm to avoid aortic insufficiency and mitigate the risk of CAVB. For all large (>6 mm) defects and cases with SAR < 3 mm, MFO was employed to prevent embolization and the development of insufficiency in the aortic and tricuspid valves. Both devices, with their soft profiles and small delivery sheaths, allow for retrograde or antegrade approaches during implantation (21, 22). The ability to be screwed from both sides facilitates the positioning and manipulation of the discs without anatomical constriction on the right side, particularly in pmVSDs with wide and aneurysmal tissue, making the MFO device advantageous compared to ADOII (23).

### Complete heart block

One of the most feared complications in percutaneous pmVSD closure is the development of CAVB. It can occur more frequently in cases where an inappropriate device is selected, and sometimes determining the exact cause of the block is challenging. Young age, low body weight, presence of ventricular septal aneurysm, selection of excessively large devices, and direct device compression are significant contributing factors (24–26). A meta-analysis of transcatheter device closure of perimembranous ventricular septal defect performed by Santhanam H et al. in 2018 showed that the pooled estimate of CAVB is 1.1% (95% CI: 0.5–1.9) (19).. In the context of pmVSD closure using the Amplatzer device, acute CAVB occurred in 2.5% of cases (within 48 h), and delayed-onset CAVB occurred in 6% of cases (between 5 and 12 months after the procedure) (27). Butera et al. reported that CAVB was more frequently observed in children under the age of 6 (28). In our series, one 13-month-old child (6.3 kg) with ADOII developed a CAVB six months postoperative and required pacemaker implantation. One 11-month-old child (5.9 kg) with MFO developed a CAVB 3 days postoperative and the device was removed. Therefore, the incidence of CAVB in our series is 4.5% for the ADOII device and 3.3% for the MFO device. In comparison with recent literature, we attribute the high incidence of CAVB in our series first to the generous device oversizing and second to the increased overall CAVB risk in the study population consisting of small more vulnerable children.

### Residual shunt

Due to the preference for MFO in large defects, residual shunts were more common in the early postoperative period in the ADOII group. In the MFO group, complete closure was achieved in 20% of cases within the first 24 h and in 96.7% of cases at 6 months of follow-up. In the ADOII group, complete closure was achieved in 9% of cases within 24 h and in 100% of cases at 6 months of follow-up. While early residual shunt rates for ADOII in the literature range from 19.6% to 71.1%, our study found lower rates of early residual shunts (6, 29). The residual shunt rates for both devices in our study were less than 5% at one year of follow-up, which is consistent with the literature.

### Valvular disturbances

The proximity of the subaortic rim, defined as the distance between the defect and the edge of the aorta, and the short distance between the septal leaflet of the tricuspid valve and the lower edge of the VSD are important risk factors in transcatheter closure (30). In cases where an ADOII device is used to prevent the development of AR and ensure a safe zone, it is recommended to have a SAR of ≥3 mm (20). In our study, we excluded defects with deficient SAR, even though it has been reported as feasible by other operators (31). The attachment point of the MFO device is flexible, allowing for the placement of the RV and LV discs at different angles. This feature facilitated seamless alignment with the defect plane in the tricuspid valve without inducing any distortion or insufficiency. In cases where the SAR (<2.5 mm) was inadequate, a transvenous approach was more frequently favored, thereby averting excessive manipulation of the wire and sheath to prevent potential harm or transient impairment to the AoV. For cases presenting aneurysmal tissue, implanting the device within the aneurysmal tissue on the RV side increased the distance between the device and the valve, consequently reducing the potential risk posed to the AoV. New-onset AR has been reported in up to 17% of percutaneously closed pmVSDs (24, 25). The development of aortic insufficiency is influenced not only by the type of device and the structure of the defect but also by the operator's experience. Experienced operators have demonstrated a lower incidence of such complications in their case series, particularly when dealing with young children (32). In our study, there was no significant increase in aortic or tricuspid insufficiency before and after the procedure.

### Limitation

There is a need for larger multicenter studies to evaluate the safety and efficacy of ADO II and MFO devices specifically in children weighing less than 10 kg. This inability to use the ADO II device for large defects during device selection led to the lack of complete equality in terms of defect size within the study population. The choice of device by the operator led to differences in the delivery approach between the antegrade and retrograde approaches. The limitations of this study include a small sample size and retrospective data collection. Conducting such comprehensive studies with a larger number of children and prospective data collection would provide consolidated evidence regarding the use of these devices in this specific children population.

## Conclusion

Both the MFO and ADOII are effective and safe closure devices in appropriately selected pmVSDs. Heart block can occur with both devices. The MFO is inherently advantageous for defects larger than 6 mm and subaortic rims smaller than 3 mm. The study results did not show any particular superiority regarding one procedural aspect, rather than defect size which is a selection bias since ADOII is not accessible for large pmVSD. Our study is the first to compare the mid-term outcomes of MFO and ADO II devices in children weighing less than 10 kg, contributing to the literature in this field.

## Data Availability

The original contributions presented in the study are included in the article/Supplementary Material, further inquiries can be directed to the corresponding author.
